# Adaptation of behavioural activation for adolescents with mild to moderate intellectual disabilities and depression

**DOI:** 10.3389/fpsyt.2026.1771276

**Published:** 2026-05-08

**Authors:** Lauren A. Cameron, Glenn A. Melvin, Richard P. Hastings, Andreas Paris, Andrew Jahoda, Amy Meade, Kylie M. Gray

**Affiliations:** 1 Deakin Centre for Child and Family Mental Health Research, School of Psychology, Deakin University, Burwood, VIC, Australia; 2 Department of Paediatrics, School of Clinical Sciences at Monash Health, Monash University, Clayton, VIC, Australia; 3 Intellectual Disabilities Research Institute, School of Social Policy and Society, University of Birmingham, Edgbaston, United Kingdom; 4 Centre for Research in Intellectual and Developmental Disabilities, University of Warwick, Coventry, United Kingdom; 5 School of Health and Wellbeing, College of Medical, Veterinary and Life Science, University of Glasgow, Glasgow, United Kingdom; 6 Department of Psychiatry, School of Clinical Sciences at Monash Health, Monash University, Clayton, VIC, Australia

**Keywords:** adaptation, adolescent, behavioural activation, depression, intellectual disabilities, psychological intervention

## Abstract

**Introduction:**

Adolescents with intellectual disabilities are at increased risk for mental health problems and depression. Despite this, there is currently no evidence for effective psychological interventions for treating low mood and depression in this population. Behavioural activation has been identified as an effective intervention for treating depression in autistic adolescents and for adults with intellectual disabilities and may therefore also be suitable for use with adolescents with intellectual disabilities.

**Method:**

The current paper describes an approach taken to adapting an existing behavioural activation intervention used with adults with intellectual disabilities (Beat-It) to be suitable for adolescents, named Beat-Depression (Beat-D). An iterative, three-phase approach was adopted for the adaptation process. The first phase involved review of the Beat-It manual and proposed adaptations by the project team, followed by a second phase consisting of consultations with parents of adolescents with intellectual disabilities and professionals with experience in the field.

**Results:**

The outcomes from phases one and two were incorporated into a final adapted manual for the Beat-D intervention. The intervention is described following the principles of the Template for Intervention Description and Replication (TIDieR) checklist.

**Discussion:**

Implications for using this adaptation approach more broadly to ensure psychological interventions used with adolescents with intellectual disabilities are suitable and accessible are discussed along with future plans for the evaluation of Beat-D.

## 1 Introduction

Adolescents with intellectual disabilities experience mental health problems at similar or higher rates than their peers without intellectual disabilities (1), with depression levels inversely associated with the severity of the intellectual disability (2). Depressive disorders experienced by adolescents with intellectual disabilities are associated with poor academic performance and loneliness (3). This increased risk of mental health concerns coupled with the additional challenges often experienced by adolescents with intellectual disabilities (e.g., communication challenges, difficulties with tasks of independent daily living) warrants the development and evaluation of specific and tailored interventions.

Currently guidelines for the treatment of depressive disorders in typically developing adolescents recommend using psychosocial therapies including cognitive behavioural therapy as first line treatments [e.g., National Institute for Health and Care Excellence (4)]. Guidelines for the management of depression for people with intellectual disabilities recommend areas of treatment delivery that may need to be adapted when working with this population. These include, for example, tailoring interventions to individual preferences, level of understanding, strengths, and needs; collaborating with the individual and their family members or carers as appropriate to identify intervention goals and structure; and providing additional structure and support (5). Despite recommendations to adapt and use psychological therapies, there is a lack of evidence supporting therapies for depressive disorders in adolescents with intellectual disabilities, exacerbated by the common practice of presence of intellectual disabilities being an exclusion criterion for participation in psychological therapy trials (6–8). Recent systematic reviews and meta-analyses seeking to establish the effectiveness of psychological therapies targeting the treatment of depression in adolescents with intellectual disabilities are limited by availability of robust studies and methodological constraints (6, 9). While there is emerging evidence for the feasibility and acceptability of psychological therapies for some mental health concerns in adolescents with intellectual disabilities, they are predominantly focused on anxiety (10), or include adolescents with co-occurring conditions such as autism (11). As such, there is no current evidence base supporting the use of any psychological therapies to treat low mood or depression in adolescents with intellectual disabilities. Appropriate and tailored interventions for mental health concerns, particularly depression, are essential in ensuring adolescents with intellectual disabilities have equal access to treatment and intervention (12). Behavioural activation (BA) is an intervention that holds promise for the treatment of depressive disorder among adolescents with intellectual disabilities. BA involves “systematically increasing individuals’ engagement in pleasurable activities … intended to bring the individual into contact with positive reinforcements in their environment” (13, p. 2). Preliminary studies, in which adult BA-approaches are adapted to suit typically developing adolescent populations, indicate some success at reducing depressive symptoms (14–16). Delivery of an adaptation of BA designed for adults with mild-to-moderate intellectual disabilities, known as Beat-It, was associated with improved depressive symptoms in a large scale randomised controlled trial (12). There are indications that BA may also be effective at reducing depressive symptoms when adapted for autistic adolescents without intellectual disabilities (17, 18). Moreover, some of the suggested adaptations for autistic people are similar to those found to be helpful for people with intellectual disabilities, such as involvement of support workers, structured sessions, and visual supports (19). However, there is currently no BA adaptation designed for adolescents with intellectual disabilities. Previous research has demonstrated that adapting existing interventions and programs for adolescents with intellectual disabilities is feasible and effective (20).

This paper describes Beat-D (Beat-Depression), an adapted BA intervention following the Beat-It (12) approach for treating depression in adults with mild-to-moderate intellectual disabilities. We describe the approach taken to tailor a BA intervention for low mood or depression to best support adolescents aged 12 to 17 years old with mild-to-moderate intellectual disabilities.

## 2 Method

We adapted an existing intervention for depression that has been successfully used with adults with mild to moderate intellectual disabilities (Beat-It). Beat-It consists of 12 sessions between a therapist, the person with intellectual disabilities, and a supporter (family member, paid support staff, friend etc.). The sessions are built upon a shared understanding of what is important to the person with intellectual disabilities and depression and take a collaborative approach to incorporating these things into the person’s life to improve their mood (12). Elements of the intervention include the use of mood thermometers (both within session and as part of ‘homework’), completion of regular activity diaries, activity scheduling and tracking, weekly reviews of progress and discussions of any barriers encountered and how they might be addressed. A formulation booklet is developed by the therapist, in collaboration with the individual and their supporter. The booklet is initially presented to the individual and supporter following the assessment phase, with discussions allowing for feedback to be incorporated into the agreed version. The formulation provides the background and focus for the therapeutic work. An updated booklet is developed for the final session, when it is handed over to the individual and their supporter. This booklet captures the progress made throughout the intervention and provides a plan for the individual and their support network to sustain and build upon the work that has been done.

An iterative, three-phase approach was taken to adapt Beat-It for use with adolescents with mild to moderate intellectual disabilities. Phase 1 consisted of a manual review and identified areas for adaptation by the project team; Phase 2 involved consultations with parents of adolescents with intellectual disabilities and professionals experienced in working with adolescents with intellectual disabilities; and Phase 3 consisted of a final revision of the intervention manual informed by the outcomes of the consultations.

Ethical approval to conduct the consultation process was received from the Monash University Human Research Ethics Committee (Project ID 19024) and the University of Warwick Humanities and Social Sciences Research Ethics Committee (HSSREC 192/21-22).

### 2.1 Phase 1: Beat-It manual review and draft adaptations

The first phase of the adaptation involved an in-depth review of the treatment manual by the project team, who are experts in the treatment of depression in adolescents (GAM) and depression in individuals with intellectual disabilities (KMG, RPH, AJ). The investigators made draft adaptations throughout the treatment manual to ensure the content was accessible for adolescents with intellectual disabilities. Initial adaptations were made across several areas including the content, setting, and delivery.

### 2.2 Phase 2: Consultations

Consultations to review the first draft of the revised manual were conducted with five parents of adolescents with intellectual disabilities, and with six professionals experienced in working with young people with intellectual disabilities. Parents (UK: n = 2 mothers, n = 1 father; Australia: n = 2 mothers) and professionals (UK: n = 2 clinical psychologists, n = 1 mental health nurse; Australia: n = 1 psychiatrist, n = 1 psychologist, n = 1 special education specialist) were recruited both within the UK and Australia to ensure the potential differences in culture and service-delivery were considered.

Consultations were conducted by a research assistant in individual or small group meetings via online videoconference and recorded. All consultations involved a presentation by the interviewer about the proposed intervention and an outline of each session was provided. Following the presentation, the research assistant asked participants a series of questions relating to: (1) overall impressions of Beat-D, including what they liked and disliked, what they believed adolescents may enjoy and/or find difficult, and what additional materials might be helpful; (2) practical aspects, including length, frequency, and number of sessions, preferences for location setting, and delivery method (i.e. online versus in-person).

### 2.3 Phase 3: Adapted manual and intervention

Participant responses and findings from the consultations were reviewed by the research team and further adaptations made to the intervention and manual.

## 3 Results

A summary of the initial adaptations made, feedback received from parents and professionals, and further revisions made addressing the feedback can be found in **Table 1**.

**Table 1:** Summary of initial adaptations, feedback received, and action taken in response to feedback.

Theme	Initial adaptations from Beat-it (adults) to Beat-D (adolescents)	Feedback received from parents/professionals	Action taken
Frequency and duration of sessions	• Sessions reduced from 60–90 minutes to 60 minutes • Inclusion of short breaks as needed	• 45–60-minute session, including breaks, recommended	• Duration of sessions revised from 60 minutes to 45–60 minutes • Recommendations to incorporate breaks as needed were included
Intervention content	• Revision of visual stimuli to be appropriate for adolescents (e.g. reflecting adolescent activities) • Revision of language to be appropriate and relevant for adolescents (e.g. school tasks instead of daytime activity) • Revision of case vignettes to reflect common challenges experienced by adolescents (e.g. problems at school) •	• Appreciation of visual stimuli • Support for inclusion of ‘jobs around the house’ domain • Offering alternative methods of completing mood diaries was suggested (e.g. an audio or recorded diary) • A number of barriers/challenges raised (e.g. screen time)	• Incorporation of alternative methods for completing mood diaries • Increased use of graphics and visual information in intervention materials • Edits to case vignettes to incorporate common challenges and barriers discussed
Delivery	• Retained qualifications/skills needed by the therapists • Retained inclusion of support person • Suggestions included for online delivery in addition to face-to-face delivery (e.g. using screensharing features)	• Reflected on the importance of the adolescent and family selecting the right supporter • Online delivery was considered feasible • Preference by parents for delivery in comfortable environments over clinical settings	• Inclusion of options for delivery of intervention online or in a place suitable to the adolescent and support person • Increased emphasis on selecting and training the support person
Context	• Description of terminology used provided (e.g. use of ‘intellectual disability’ in place of ‘learning disabilities’ • Removal of region specific terminology (e.g. ‘hoover’ versus ‘vacuum) • Consideration of service delivery contexts	• No feedback specific to the UK or Australian context received • Parent and professional views did not differ based on where they were located	• None

### 3.1 Phase 1: Beat-It manual review and initial adaptations

Following the initial review of the treatment manual, the following elements were adapted for suitability with an adolescent population.

#### 3.1.1 Content

Within the introduction to the manual, the literature was revised to reflect the current evidence base regarding the presentation and treatment of depression in adolescents. Throughout the manual, the language was updated to reflect an adolescent population. This included examples of appropriate activities (e.g., playing sport, seeing friends, doing homework), References to the intervention supporter to facilitate the adolescent’s participation in the intervention (i.e., the inclusion of parents), and relevant clinical information (e.g., possible factors that may impact the intervention such as behaviours that challenge).

Further, the language used within the delivery of the treatment itself was revised to be more developmentally appropriate for adolescents. For example, the Beat-It intervention is designed to increase activity across three main domains: (1) domestic or household activity, (2) purposeful daytime activity, and (3) social/leisure activity. For Beat-D the following changes were proposed to better suit adolescents: (1) house tasks/chores, (2) school tasks (academic/social), and (3) after school and weekend leisure/social activities.

References to life goals and values were considered likely to be difficult for some adolescents to understand or articulate. Language was changed to highlight these elements as ‘things that are important to the adolescent’.

All resources and materials, including parent and adolescent handouts, the Mood and Activity Diaries, and session resources (e.g. mood and feelings thermometers, activity tracking sheets), were redesigned to be appropriate and appealing for an adolescent audience and incorporate the revised language. This included, for example, ensuring images depicted adolescents engaged in activities and tasks, formatting of checklists and worksheets for consistency and clarity, and, where possible, using language at an appropriate reading level for adolescents with mild intellectual disabilities.

Case vignettes provided in the manual appendices to describe working with barriers to change that may be encountered throughout therapy, were re-written to reflect common challenges amongst adolescents and appropriate strategies for managing these challenges. Adolescents may be experiencing anxiety, significant anger or behavioural challenges, or problems at school that are impacting their ability to engage with Beat-D for depression and low mood and are therefore a barrier to the success of the intervention. These vignettes describe these challenges and present some strategies the therapist may find helpful in addressing them. For example, one vignette described how anxiety might present in an adolescent and how the therapist could use the strategies within Beat-D to address this.

#### 3.1.2 Delivery

Beat-D has been designed to be delivered by someone who is experienced in working with young people with intellectual disabilities and mental health problems. This person is referred to as the therapist throughout the Beat-D manual. The therapist should have some level of qualification in the field of mental health and/or intellectual disabilities, for example, a school social worker or wellbeing staff, occupational therapist, or mental health nurse. Specific training for the therapist in the delivery of the intervention, with a focus on ensuring delivery is developmentally appropriate for the adolescents undertaking the intervention, along with regular supervision, was retained from the original Beat-It manual.

A key element of Beat-It is the involvement of a supporter (e.g., family member, paid support person) whose role is to provide additional support to the individual receiving the intervention both within sessions and outside in their day-to-day life. It was considered important for the supporter to be someone who knows the adolescent well and is available to support the adolescent outside of the sessions. The proposed adaptations for Beat-D retained the involvement of the supporter as an essential part of the intervention process. Individual time with the therapist was included to allow for the adolescent to work with the therapist individually if desired, recognising that some adolescents may have things they feel uncomfortable talking about in front of a family member or other support person. This is initiated by the therapist when setting the agenda by asking the adolescent if they would like to make time during the session to talk together alone.

The manual review and adaptation took place during the early stages of the COVID-19 pandemic and therefore adaptations for online administration were important. This included incorporating examples of how activities might be run via videoconferencing, for example, using the whiteboard feature on Zoom, and sending out physical copies of resources prior to sessions. This was particularly relevant for Session One, with specific details included in the manual on how the activities during this session could be undertaken either online or in-person. For example, the Post-Box task requires the individual to select printed images of various activities and ‘post’ them in boxes according to i) things they currently do, ii) things they used to do, and iii) things they would like to try. A suggested adaptation for online delivery included displaying the images on a PowerPoint presentation and using the screen sharing and whiteboard feature on Zoom to allow the individual to indicate which activities might belong in each category.

To reduce the impact of fatigue for adolescents, session length was reduced to 60 minutes (as opposed to 60–90 minutes for the adult population), in addition to the inclusion of short, 5-minute breaks as needed.

#### 3.1.3 Context

The Beat-D adaptation was designed to be used in both the UK and Australia and potentially other international settings. In most instances, language was used that would be appropriate for either context. However, there were some differences in language and terminology across contexts that required further clarity. For example, the term ‘learning disabilities’ is widely used throughout the UK in place of ‘intellectual disabilities’. Therefore, a new section is included in the manual describing the terminology to be used.

### 3.2 Phase 2: Consultations

Parent and professional responses were categorized into five key themes, consistent with the structure of the interview questions: frequency and duration of sessions, intervention content, supporter, delivery mode, and barriers and challenges.

#### 3.2.1 Frequency and duration of sessions

Both parents and professionals believed 60 minutes would be the longest an adolescent with intellectual disabilities would be able to maintain attention, with recommendations for sessions being 45–60 minutes, with breaks necessary.

##### 3.2.1.1 Parents

The length of the sessions (60 to 90 minutes in the original Beat-It manual) was identified as a possible barrier to attendance and engagement by parents, with some indicating this would not be possible for adolescents with a short attention span.

*“He’s very active and he’s always running around, moving around, can’t sit still that long so … definitely would need breaks but one hour would be good timing.” [Parent 1, Australia].*

Parents suggested increasing the number of sessions and reducing the length of each one. The importance of providing breaks throughout each session was also highlighted by all parents, with one parent suggesting that the therapist should ensure they regularly check in with the adolescent in case they do not ask for breaks when they need it.

Parents noted that the length of time between sessions should be tailored to the individual as some adolescents might require more or less frequent sessions.

*“Some children might need more encouraging, they might need two sessions a week, some kids might only need one session a week, some kids could probably go once a fortnight.” [Parent 2, Australia].*

##### 3.2.1.2 Professionals

The 12-week duration of the intervention yielded some mixed responses from professionals. While some suggested 12-weeks would likely be too short to see change and may not be enough time for adolescents to engage in activities set out as goals, others indicated that a 12-week intervention was likely sufficient based on their prior experience.

The 60 to 90-minute sessions was considered to be too long by most professionals, with a 45 to 60-minute session suggested as more suitable. The importance of including breaks throughout the session was highlighted although some noted this might also require additional steps in re-engaging the adolescent after a short break.

*“I wonder about one and a half hours duration, particularly if you’re thinking about kids with mild intellectual disability … I would think that an hour and a half is a long time for them to engage in the session. … more likely 45 minutes to an hour is going to be more comfortable…” [Professional 1, Australia].*

The inclusion of an optional booster session was also suggested.

#### 3.2.2 Intervention content

Both parents and professionals agreed that the inclusion of visual stimuli wherever possible would be essential to adolescent engagement and understanding. Parents were interested in the elements that would support the adolescent to engage independently wherever possible, while professionals noted the importance of the post-intervention supports such as the formulation booklet.

##### 3.2.2.1 Parents

Incorporating as much visual stimuli as possible was emphasised as important in enhancing the adolescent’s engagement and understanding. This extended to the homework tasks, with parents suggesting the inclusion of visual checklists.

*“He’s very visual so only pictures would be ideal with him.” [Parent 1, Australia].*

There was strong support for the inclusion of the ‘jobs around the house’ domain and parents considered this especially important in increasing daily living and self-help skills. Parents also commented that this would be beneficial for the adolescents in increasing their sense of responsibility.

The importance of giving the adolescent ownership and responsibility over the intervention, and the positive impact that would likely have on engagement, was noted by multiple parents.

*“I like the working towards change … to get them involved … [child] needs responsibility.” [Parent 2, Australia].*

*“And the idea of them taking ownership of it … brilliant. My daughter is much more likely to stick at something if she feels it’s her plan, her goals.” [Parent 3, UK].*

The activity and mood diary was considered a positive element, although it was suggested that having the option of completing an audio or recorded diary might be more feasible and helpful for some adolescents who are unable to write. This was suggested as a way of maintaining control over the diary rather than relying on another person to write for them if unable.

*“I think that the diary, this might be helpful. Like my son, who still can’t write to standard, a recorded diary … because that would make him feel more comfortable … and he doesn’t feel pressured to have to write it and get frustrated.” [Parent 2, Australia].*

*“Could we have audio options too? Like record your mood instead of writing? My daughter loves recording voice notes … she feels proud playing them back.” [Parent 4, UK].*

*“Make it accessible. And maybe even give them the choice … voice, pictures, or words … so it feels like their project.” [Parent 5, UK].*

##### 3.2.2.2 Professionals

The use of images, graphics, and succinct language throughout the intervention and the resources was identified as a feature that will be helpful in aiding the understanding and participation by the adolescents.

*“I think there needs to be as much graphic information as possible. I think that when you’re talking about concepts of mood and feelings … you need to be able to show them … to make it clearer.” [Professional 1, special education specialist, Australia].*

*“I can see a young person feeling “this is for me” rather than “this is another boring adult thing.”“ [Professional 4, UK]*.

The importance of completing the mood diary in close proximity to the completion of the activity in an engaging way for the adolescent was noted, with suggestions that the therapist and supporter should encourage the young person to complete the diary after each activity where possible rather than reviewing and completing at the end of the day.

*“I think it would be probably more accurate if you’re doing it as close to the time of the activity as possible rather than thinking about recall.” [Professional 1, Australia]*.

*“Use smiley faces, colours, stickers … whatever they find quick and fun.” [Professional 4, UK]*.

The formulation booklet was identified as an excellent tool for providing ongoing support to the adolescent, particularly for the parts of their life that the supporter is not directly involved with, for example, providing the booklet to teachers to help them understand what the young person’s goals are.

*“I really liked the idea of the booklet because it means that the end of it, the child can look back over what’s happened, what they’ve achieved, and so on.” [Professional 1, Australia].*

*“It’s an excellent idea to give young people a concrete, visual map of what they are working on.” [Professional 5, UK].*

One professional noted that it would be helpful to break down short, medium, and long-term goals and ensure it is highlighted to the adolescent and supporter that more complicated, long-term goals are unlikely to be achieved by the end of the 12-week intervention. Starting the sessions with smaller and more easily achievable goals and activities will be important to quickly see some positive change and progress.

*“We always make sure that we start with something which is more easily achieved so that they’re successful and it builds confidence.” [Professional 1, Australia].*

#### 3.2.3 Supporter

The importance of selecting the right supporter was recognised by both parents and professionals. Parents considered elements that would impact the support person’s engagement and enjoyment (e.g., who they enjoyed spending time with), while professionals highlighted the importance of ensuring the supporter had the necessary skills and availability to provide support.

##### 3.2.3.1 Parents

The importance of involving parents at the beginning stages of the intervention was emphasised as the intervention is likely to affect the whole family. They noted that it would be important for the therapist to make sure parents are involved and their views on their adolescent’s interests and possible activities would be considered.

*“That would be a good stage for someone like me … to put in my input because this is going to affect my family, me, [my child], so I’d need to know what [child’s] interested in getting support on and changing and to be kept in the loop…” [Parent 2, Australia].*

*“I think discussing it beforehand is key … and maybe being flexible if it changes once they start.” [Parent 5, UK].*

Further, the selection of the supporter was identified as a critical step and discussion around this would be essential prior to commencement of the intervention. While some parents thought that their adolescent would prefer a support worker to be involved rather than a parent, others commented that the adolescent would probably feel more comfortable if a parent was involved. Another parent commented that they (the parent) would feel uncomfortable if they were not there as they would not know what is happening in the sessions and would not be able to clarify what the adolescent is saying.

*“I know he likes one of the carers that he goes out with, he really does trust …” [Parent 2, Australia].*

There was a preference for a carer or support worker to be involved in the sessions in some instances as adolescents are often used to trying new activities with their support staff, although the practical difficulties in terms of extra costs and planning in involving the supporter, difficulties in the generalisation of learned skills, and instances where the adolescent wanted activities with the parent, was raised.

*“He would probably get less resistance if he had one of his carers, but the problem with the carers, they don’t live here 24/7…” [Parent 2, Australia].*

Bringing additional people into the final sessions was suggested as something that would be beneficial as it would allow more people in the adolescent’s life to gain an understanding of what has occurred in previous sessions and what future plans involve.

##### 3.2.3.2 Professionals

The role of the supporter was recognised as key to the achievement of long-term goals, including upskilling the supporter in providing ongoing support with the program strategies beyond the end of the intervention period.

*“… component of teaching the key carer about behavioural activation so that it can be continued. That is the key component.” [Professional 3, Australia].*

Professionals also acknowledged the barriers that could arise for the supporter, including a need to focus on their own problems, and other time commitments, and noted that they would need to engage in their own supports, as appropriate.

#### 3.2.4 Delivery mode

Adaptations to deliver the intervention online, when in-person delivery was not possible, were proposed. Online delivery was considered feasible by parents and professionals, although the individual’s cognitive ability and attention span were highlighted as considerations that may impact this. When delivering in-person, professionals believed a community-based or clinical setting would be most appropriate while parents considered these settings to be too clinical and preferred a more comfortable environment. The parents’ views were consistent with the original Beat-It intervention, which is delivered on an outreach basis, where the person lives. The underpinning logic is that the therapist would gain a more tangible insight into the person’s everyday life, the opportunities for activity and barriers to change.

##### 3.2.4.1 Parents

Relaxed and less formal environments were noted as preferable to hospital environments for in-person delivery. For example, one parent described that their adolescent enjoyed attending their psychologists’ office in an old residential building, rather than clinical or hospital environments.

*“He doesn’t really like going into hospitals, like that sort of environment, he’s not big on that.” [Parent 2, Australia].*

Online delivery was seen as a positive element in terms of encouraging adolescent engagement. Parents reported previous positive and successful experiences with online programs during COVID-19 lockdowns. In many cases, parents thought online would be easier for the adolescent to engage in, especially considering the difficulties they are likely to have engaging in activities in person.

Online delivery was noted as potentially problematic for adolescents with moderate intellectual disabilities, with one parent stating this would not be possible for their adolescent.

*“He wouldn’t cope with things online … he really needs to be there to be able to know what’s going on.” [Parent 1, Australia].*

Multiple parents noted that, while their adolescent had recently taken to engaging in more online groups, they still desired personal interaction, and therefore finding a balance between online and in-person would be important.

*“He likes engaging online …. I think he really, he wants personal interaction as well…” [Parent 2, Australia].*

*“He likes online for comfort but does miss real-world contact. I reckon a blended model would be ideal … mostly online, but maybe a few in-person options when possible.” [Parent 5, UK].*

##### 3.2.4.2 Professionals

The online delivery mode was well accepted by professionals, with most indicating this would be a preferred delivery method. Professionals referred to their experience in delivering interventions online, particularly during COVID-19 lockdowns, which had been successful.

If being delivered in-person, one professional suggested a clinical setting would be most acceptable to adolescents as, in their experience, adolescents often do not want to be taken out of class as it makes them feel different, while the home environment is likely to pose further distractions.

Home distractions were identified as a possible problem with online delivery, with professionals highlighting the importance of the role of the supporter in supporting the adolescent to be engaged. It was suggested that guidelines be provided for the supporter, including finding a quiet room or space in the house to participate in the sessions and appropriate methods for encouraging engagement (i.e. avoiding extrinsic motivators, disapproval, or coercive behaviours).

*“It allows for shorter bursts of work, use of visual aids easily on-screen, and flexibility for families who might struggle with transport.” [Professional 5, UK].*

All professionals indicated that the session activities would translate well online although some supplementary supports might be helpful. For example, the ‘post-box’ task in session one (a task in which the adolescent sorts images of various activities into (1) things I used to do, (2) things I used to do but stopped, and (3) things I would like to try) could be delivered effectively online with the use of images and boxes in a presentation that is shared with the participants. Alternatively, mailing physical copies of images to the family prior to the session for the adolescents to work with was possible, if preferred. Having multiple options for completing tasks based on adolescent preference (e.g. an online and a paper-based option) was noted as an important consideration.

*“…physically send them something … so they had something written down, pictures that they could hold or look at.” [Professional 2, Australia].*

#### 3.2.5 Barriers and challenges

Parents noted potential barriers relating specifically to the types of activities that their adolescent might engage in, while professionals highlighted the potential impact of co-occurring diagnoses and challenges that may impact activity engagement.

##### 3.2.5.1 Parents

Difficulty in engaging adolescents with moderate intellectual disabilities was noted as a potential barrier, particularly in regard to sustained attention via the online format.

*“So many things play into it, how his day is going, what else he’s been doing, how tired he is to cooperate.” [Parent 1, Australia].*

*“Short, snappy, and interactive. Otherwise their brains just drift.” [Parent 5, UK].*

It was noted that parents felt they would struggle with knowing what types of activities they could suggest for their adolescent and how they would assess if the adolescent really is enjoying them. Further, parents wondered if the intervention would be more effective for adolescents who are more willing to and already engage in different activities.

##### 3.2.5.2 Professionals

Professionals noted multiple barriers and challenges to the adolescent’s participation in the program. Adolescents’ screen time was identified as a potential challenge to engagement in BA activities, especially in the context of gaming addiction and increased screen time reinforcing withdrawal behaviour, with suggestions to track adolescents ‘other’ screen activities. Further, assessment of comorbidities (e.g. ADHD, anxiety, school refusal, gaming addiction) that may impact intervention needs to be considered. The delivery and effectiveness of the intervention for adolescents with these more complex presentations was considered likely to be more difficult and something that will need to be considered. This will be particularly important to address for adolescents with school refusal given one of the target domains of activity includes activities at school.

Professionals noted that some adolescents may be reluctant to change their behaviours and therefore the initial sessions would be critical in ensuring the adolescent feels empowered and part of the change process.

*“The patient has to feel empowered and they are a part of the change process because … often when we are talking about any change that can cause a massive amount of anxiety.” [Professional 3, Australia].*

Financial barriers for families was identified as a challenge likely to be encountered (e.g., transport costs, activity participation costs such as sporting club fees or uniforms, additional costs for engaging a support worker) and providing guidance for the therapist in how to manage this was suggested for inclusion.

*“It costs nothing to go for a walk but you know, might cost quite a lot to join to go to a swimming pool … have someone to go with you, to get the transport …” [Professional 2, Australia].*

*“Normalising low-cost or free activities from the start? Walking, visiting parks, cooking,… these are all valid and powerful activations.” [Professional 5, UK].*

### 3.3 Phase 3: Adapted manual and intervention

Feedback from the consultations led to a number of further adaptations to Beat-D. These included:

A description of the adapted intervention (i.e. Beat-D) is described below following the elements of the TiDieR checklist (21).

#### 3.3.1 Intervention name and rationale

The final intervention, Beat-Depression (Beat-D), aims to improve symptoms of low mood or depression in adolescents aged 12 to 17 years with mild and moderate intellectual disabilities through increasing engagement in meaningful activities across three areas of life: household tasks/chores, school tasks, and after school and weekend leisure/social activities, following the principles of BA.

#### 3.3.2 Description of intervention materials and procedures

A Beat-D therapist manual was developed based on the previous Beat-It manual, detailing the intervention and materials and resources required. The manual includes:

- detailed session plans describing the structure of each intervention session, including the activities to be undertaken and checklists for the therapist,
- a therapy information leaflet that is provided to the adolescent prior to commencing Beat-D, introducing them to the intervention and what they can expect,
- a supporter presentation, which the therapist should follow in their initial session with the supporter prior to bringing in the adolescent,
- a mood ratings sheet, used at the beginning of each session to support the adolescent in identifying their current mood,
- a Mood and Activity Diary, provided to the adolescent during the first session, which they will complete each day with the help of their supporter in a method they feel comfortable with (e.g. written, audio recording),
- a recording sheet for the Post-Box Task in session one,
- templates for activities in session one to capture the adolescents:
  - life goals and values (“Things that are important in my life”)
  - future goals (“Things I want for my future”)
  - things that bring joy (“Things that make me smile”)
  - wishes (“My three wishes”),
- ‘Make a plan’ template, used during the session to plan the activity the adolescent will attempt that week,
- ‘How did it go?’ template, used during the session to reflect on the weekly activity and identify any barriers to engaging in the activity,
- an ‘activity hierarchy’ template, used to capture the activities that the adolescent identifies in session one,
- a ‘certificate of completion’, presented to the adolescent at the end of their final session,
- a formulation booklet template, used by the therapist to develop the initial formulation booklet for review and feedback with the adolescent and supporter.
- vignettes describing challenges that a young person might face in addition to low mood and guidance for managing these where they are interfering with the young person’s engagement in the behavioural activation intervention.

See **Table 2** for a description of the aim and activities for each session.

**Table 2:** Overview of Beat-D sessions.

Session	Aim	Activities
1	Getting to know you	• Explain the purpose of the intervention • Determine what activities the adolescent currently does, has done in the past, and would like to do • Explain how to monitor mood and activity
2	Identify targets and goals	• Review activity and mood from previous week • Identify targets and goals for intervention • Introduce first trial of home activity
3	Review and plan activities	• Review home activities • Plan activities
4	Review progress and identify barriers	• Review progress – have planned activities been completed? • Identify barriers to progress
5	Introduce formulation booklet	• Present and explain formulation booklet
6 - 10	Continue with planned activities	• Continue with planned activities • Address individual and systemic barriers to change • Introduce new activities as appropriate
11	Review of progress	• Review progress • Identify activities that were most helpful and how they can be continued in the long term
12	Review of progress and present final formulation booklet	• Review of progress to date • Present final formulation booklet

Beat-D includes several design features that aim to engage the adolescent and provide structure to the intervention. These include:

#### 3.3.3 Who delivers the intervention

Beat-D is designed to be delivered by people who work with adolescents with mild to moderate intellectual disabilities and who have general knowledge of, training, and experience with, supporting young people with mental health concerns. This might include wellbeing support staff in school settings as well as in health services under the supervision of a trained or experienced clinician.

#### 3.3.4 How the intervention is to be delivered

The intervention has been designed to be delivered either face-to-face or online.

#### 3.3.5 Where the intervention is delivered

Online delivery allows the adolescent and their supporter to participate in the sessions at a place that is convenient to them. They should select somewhere quiet and private, for example, a separate room or space in the home with minimal distractions. Face-to-face sessions may be delivered in a location that is suitable for the adolescent, such as their school or the therapist’s clinic.

#### 3.3.6 Length and duration of the intervention

Beat-D consists of 12 weekly sessions, with an optional booster session available. Each session runs for 45–60 minutes and allows for breaks to be taken as needed by the adolescent.

#### 3.3.7 How the intervention can be tailored

The intervention focuses on working with the adolescent and their supporter to increase activity in a range of areas. The types of activities are discussed with the adolescent and supporter, and tailored to be specific to the individual’s desires and how feasibly they can be incorporated into the individual’s routine. For example, cost, time, and availability may prohibit some activities being suitable.

The formulation booklet is developed throughout the intervention and provided to the adolescent upon completion of the intervention. It is specifically tailored to the individual to capture the progress made and provide information on the activities that worked for the individual.

Any co-occurring conditions, such as autism, ADHD, and anxiety, or additional challenges experienced by the young person are considered by the therapist. The intervention can be further tailored to address additional needs, including, for example, communication preferences, timing of sessions, and suitability of suggested activities.

#### 3.3.8 How the intervention was adapted for adolescents

The initial modifications made to this intervention for suitability with the target population (i.e., adolescents with mild to moderate intellectual disabilities) are described in section 3.1, with further adaptations following consultation with parents and professionals described in section 3.3.

#### 3.3.9 How the intervention will be assessed

The adolescent’s progress throughout the intervention is monitored and assessed through weekly review of the Mood and Activity Diary. The therapist will draw the adolescent’s attention to changes in activity engagement and corresponding changes in mood, highlighting positive progress made and reviewing barriers and challenges where improvements in mood are not evident.

## 4 Discussion

The increased risk of mental health concerns, particularly depression, coupled with a lack of evidence-based psychological interventions for adolescents with intellectual disabilities, highlighted a significant gap in the provision of mental health care for this population. Taking an existing approach that has been demonstrated to be effective for adults with intellectual disabilities (12) and adapting it to be suitable for adolescents is an efficient way to address this gap and develop an intervention that can be delivered to adolescents with depression. This paper describes the process of adapting a BA intervention for depression for adolescents with intellectual disabilities, with a focus on ensuring the intervention is suitable and accessible for this population. Importantly, while this adaptation process was specific to BA, key elements are likely to be helpful when considering the adaptation of any psychological intervention for adolescents with intellectual disabilities.

Throughout the adaptation process, the expertise of families, clinicians, and researchers was drawn upon to ensure the adapted intervention, Beat-D, would meet the needs of the adolescent and their families. The wider impact on the family, including parents’ time, resources, finances, and the needs of siblings and other family members, was necessary to consider when adapting the intervention from targeting an adult population to an adolescent population, with increased demands and involvement from the adolescent’s parents and family members likely.

Key adaptations were made to the Beat-It intervention to ensure suitability and acceptability to adolescents. These included: reduction of intervention time from 60–90 minutes to 45–60 minutes; use of developmentally appropriate language and including examples throughout the manual, the intervention content, and the intervention materials and resources. Moreover, increased use was made of visual stimuli and graphic presentation of information. Increased recognition of the role of the supporter was evident with the importance of discussion between therapist, family, and adolescent at the beginning of the intervention regarding choosing who the most appropriate person for this role should be. Importantly, the adaptations made to Beat-It, resulting in the Beat-D intervention for adolescents, maintained the core elements of the intervention, including the session structure, content, and activities (e.g. setting homework tasks to try out an activity, weekly reviews of how activity plans went, development of the formulation booklet) as well as the involvement of the supporter.

The consultations with parents and professionals revealed largely consistent views on the proposed intervention. Both groups discussed that the session length needed to consider the concentration ability of the adolescent, the importance of the inclusion of visual information, and the integral role of the supporter. The main area where parents and professionals differed in opinion related to the location of in-person intervention delivery. While professionals felt that a hospital or clinic provided a distraction-free environment, parents suggested that adolescents may find these types of environments more clinical and uncomfortable and prefer a more relaxed environment. It would be necessary for the therapist delivering the intervention to discuss the location with the family upon intake, keeping in mind their own workplace policies and limitations and the requirements of the family to ensure the adolescent is engaged in the intervention.

The approach taken in adapting an intervention for adults with intellectual disabilities for an adolescent population with intellectual disabilities was consistent with that seen in non-intellectual disabilities populations. For example, Lewinsohn et al. (22) adapted the Coping with Depression course originally designed for use with adults, for adolescents. In their adaptation, they retained the core elements of the therapy and modified the content to be developmentally suitable for adolescents and included additional adolescent-focused topics such as parent-adolescent conflict resolution. Similarly, Pass et al. (14) describe their adaptation of BA from a typically-developing adult to a typically-developing adolescent population. They kept the core intervention content consistent but modified the manual to be better suited for adolescent clients, incorporated adolescent specific case examples to demonstrate the key concepts of the intervention, and invited parents to attend specific sessions throughout the intervention.

### 4.1 Future directions

Before it can be implemented into practice, Beat-D now needs to be thoroughly evaluated ideally in collaboration with either clinical and/or school wellbeing services. Initially a modelling and/or feasibility study is warranted, and if the intervention is found to be feasible and acceptable, a definitive randomized controlled trial would then be undertaken. Involving people with intellectual disabilities and their families in this evaluation is essential.

### 4.2 Strengths and limitations

The adaptation of Beat-D was strengthened by the involvement of parents and therapists from a range of disciplines from across two countries. It is acknowledged that this adaptation would have been strengthened by the inclusion of the opinions of adolescents with intellectual disabilities. Future evaluations of Beat-D are planned and will involve gathering the views of young people with intellectual disabilities on the intervention and how it might be improved further to meet the needs of young people.

### 4.3 Conclusion

Beat-D is a promising adolescent adaptation of the evidence-based Beat-It BA-based intervention for adults with depression. Beat-D has the potential to fill a gap by providing a suitable intervention for adolescents with intellectual disabilities and depression in an environment where currently no evidence-based treatments are available. Future research is now required to comprehensively evaluate the effectiveness of Beat-D in rigorous clinical trials.

## Data Availability

The datasets presented in this article are not readily available due to participant confidentiality. Requests to access the datasets should be directed to Kylie Gray, k.m.gray@bham.ac.uk.
